# Tyrosine phosphorylation regulates ERβ ubiquitination, protein turnover, and inhibition of breast cancer

**DOI:** 10.18632/oncotarget.10018

**Published:** 2016-06-14

**Authors:** Bin Yuan, Long Cheng, Kshama Gupta, Huai-Chin Chiang, Harshita B. Gupta, Gangadhara R. Sareddy, Degeng Wang, Kate Lathrop, Richard Elledge, Pei Wang, Stanton McHardy, Ratna Vadlamudi, Tyler J. Curiel, Yanfen Hu, Qinong Ye, Rong Li

**Affiliations:** ^1^ Department of Medical Molecular Biology, Beijing Institute of Biotechnology, Collaborative Innovation Center for Cancer Medicine, Beijing, China; ^2^ Institute of Cancer Stem Cell, Cancer Center, Dalian Medical University, Liaoning, China; ^3^ Department of Molecular Medicine, University of Texas Health Science Center at San Antonio, San Antonio, TX, USA; ^4^ Department of Medicine, University of Texas Health Science Center at San Antonio, San Antonio, TX, USA; ^5^ Department of Obstetrics and Gynecology, University of Texas Health Science Center at San Antonio, San Antonio, TX, USA; ^6^ Department of Epidemiology and Biostatistics, University of Texas Health Science Center at San Antonio, San Antonio, TX, USA; ^7^ Department of Cellular and Structural Biology Cancer Therapy and Research Center, University of Texas Health Science Center at San Antonio, San Antonio, TX, USA; ^8^ Center for Innovative Drug Discovery, University of Texas at San Antonio, San Antonio, TX, USA

**Keywords:** tyrosine phosphorylation, ERβ, ubiquitination, antitumor activity, protein turnover

## Abstract

Unlike estrogen receptor α (ERα) that predominantly promotes hormone-dependent breast tumor growth, ERβ exhibits antitumor effects in a variety of cancer types. We recently identified a phosphotyrosine residue in ERβ, but not ERα, that dictates ERβ transcriptional activity and antitumor function. We show here that this ER isotype-specific phosphotyrosine switch is important for regulating ERβ activity in cell proliferation, migration, and invasion. At the mechanistic level, phosphorylated ERβ, which recruits transcriptional coactivator p300, is in turn targeted by p300 for ubiquitination and proteasome-dependent protein turnover. Furthermore, ERβ-specific agonists such as S-equol enhance ERβ phosphorylation, suggesting a crosstalk between ligand- and posttranslational modification-dependent ERβ activation. Inhibition of xenograft tumor growth by S-equol is associated with reduced tumor Ki-67 expression and elevated ERβ tyrosine phosphorylation. Taken together, our data support the notion that phosphotyrosine-dependent ERβ signaling is an attractive target for anticancer treatment.

## INTRODUCTION

The diverse physiological and pathological effects of estrogens are mediated by two estrogen receptors, ERα and ERβ, which are encoded by different genes (*ESR1* and *ESR2*) [1]. Although these two ER isotypes share homologous protein sequence and similar transcriptional activity, they exhibit quite distinct biological functions in cancer development and progression. ERα is well documented for its role in promoting estrogen-dependent breast tumorigenesis, whereas ERβ has been reported to inhibit tumor growth in multiple cancer types including breast and ovarian cancers, melanoma, and glioma [1–7]. In addition to common target genes shared by these two ER isotypes, ERβ binds to its own transcriptional target genes through either estrogen response elements (ERE) or by tethering to other DNA-binding transcription factors [8–19]. The ERα-independent activity of ERβ represents a prevailing mode of ERβ action in ERα-negative cancers. In ERα-positive cancer cells, ERβ is also capable of interfering with ERα activity through hetero-dimerization and/or competition for common binding sites [6, 14, 15, 20–30], thus making ERβ a partial dominant negative receptor for ERα [23, 24, 31]. Clearly, ERβ functions in transcription and cancer are different from those of ERα.

The disparate functional outcomes of the ERα and ERβ actions are at least partly due to differences in protein structure between these two ER subtypes. Despite a highly homologous central DNA binding domain (DBD, 96% identity) and carboxyl (C)-terminal ligand-binding domain (AF2 + LBD, 53% identity), the amino (N)-terminal sequence (AF1, 18% identity) is quite divergent between these two ER subtypes. This AF1 domain has been linked to subtype-specific transcription activity of ERβ [32, 33]. Furthermore, we recently discovered that an ERβ-specific phosphotyrosine residue in the AF1 domain of ERβ (pY36) dictates the antitumor activity of ERβ [34]. This tyrosine residue is highly conserved in all mammalian ERβ orthologs, but not in ERα (alanine in ERα). Our published work shows that mutation of Y36 to phenylalanine (Y-F) obliterates ligand-dependent transcription and ERβ antitumor activity in ERα-negative breast cancer [34]. Of note, ERβ phosphorylation status strongly correlates with longer survival in breast cancer patients [34]. Thus, this newly identified phosphotyrosine switch offers a potential opportunity to fine-tune ERβ biological activity in cancer with precision and potency.

In the current work, we investigated the influence of the phosphotyrosine switch on tumor cell proliferation, migration, and invasion. In addition, we determined how abundance of phosphorylated ERβ protein was regulated in cancer cells. Finally, we found that the ERβ-specific agonist S-equol induced ERβ phosphorylation and inhibited tumor growth *in vitro* and *in vivo*. Our study lends further support to the notion that turning on the phosphotyrosine switch in ERβ is important for mobilizing its antitumor activity.

## RESULTS

### The phosphotyrosine switch is important for antiproliferative activity of ERβ

**Figure 1:**
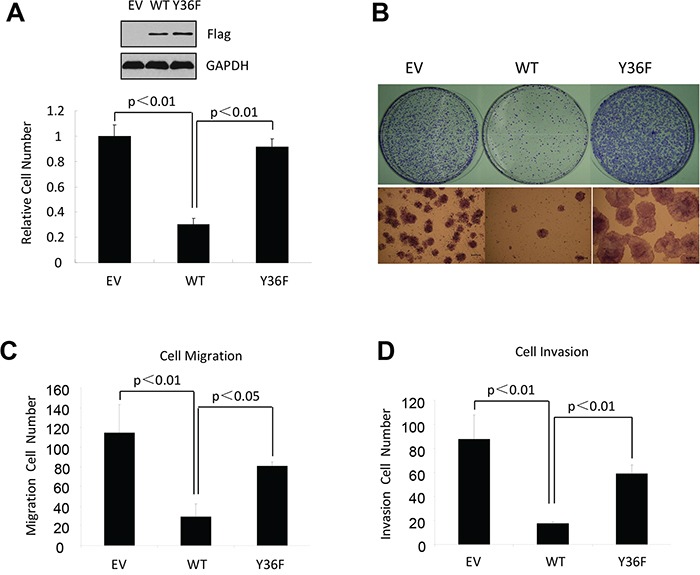
The phosphotyrosine switch is important for ERβ activity in multiple cancer types **A.** *In vitro* MTT assay using MCF7-derived breast cancer cells that contained empty vector, WT, or Y36F mutant ERβ. Western blot of Flag-ERβ proteins in MCF7 stable cell lines. **B.** WT but not Y36F mutant ERβ overexpression in MCF7 cells reduced colony formation. **C.** and **D.** WT but not Y36F mutant ERβ overexpression reduced cell migration (C) and invasion (D). Data here and elsewhere represent average of at least three biological duplicates. Error bars indicate s.e.m.

Our published work demonstrates that the ERβ phosphotyrosine switch regulates antitumor activity of ERβ in triple negative breast cancer (ERα^−^/PR^−^/HER2^−^) [34]. To determine the generalizability of our previous conclusion, we introduced both wild-type (WT) ERβ and the functionally inactive tyrosine-to-phenylalanine (Y36F) mutant into ERα-positive breast cancer cells (MCF7), ovarian cancer cells (SKOV3), and glioblastoma cells (U87), which represent three cancer types where ERβ antitumor activity had been previously demonstrated [1, 4, 35]. Consistent with the previous reports, WT ERβ significantly reduced tumor cell viability in all three tumor cell lines (Figure 1A–1B, Supplementary Figure 1). In contrast, cells expressing the Y36F mutant exhibited little suppression of cell viability, suggesting that the phosphorylation switch is required for ERβ antiproliferative activity in multiple tumor cell types. In addition to cell proliferation, ERβ can inhibit cell migration and invasion [36]. In this regard, we found that ERβ-mediated inhibition of both tumor cell migration and invasion were significantly compromised by the Y36F mutation in MCF7 cells (Figure 1C–1D), thus implicating a role of the phosphotyrosine switch in multiple aspects of ERβ-mediated antitumor function.

### pY36 facilitates coactivator-dependent ubiquitination and turnover of ERβ

**Figure 2:**
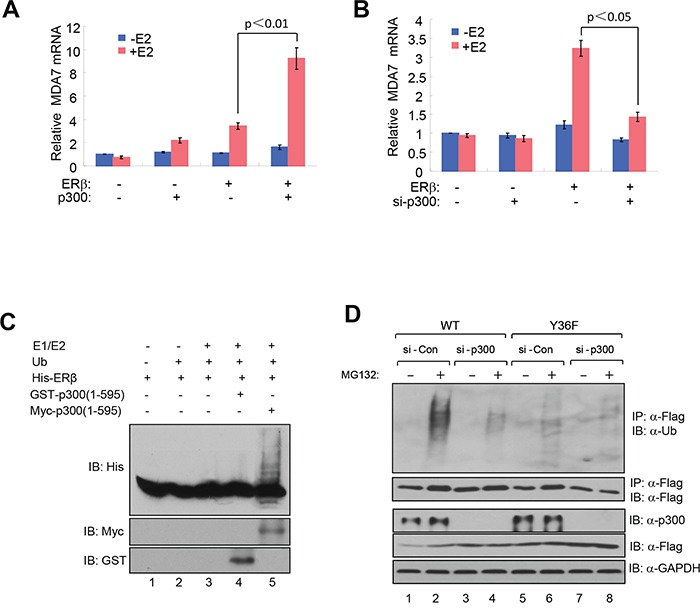
pY36 facilitates coactivator-dependent ubiquitylation and turnover of ERβ **A.** Ectopic expression of p300 promoted ligand-dependent transcriptional activation of MDA7 by ectopic ERβ in HEK293T cells. **B.** siRNA-mediated p300 knockdown blunted ectopic ERβ activity in HEK293T cells. **C.** *In vitro* ubiquitination assay containing E1/E2 ubiquitin ligases, purified bacterially expressed His-ERβ, bacterially expressed GST-p300(1-595) or mammalian cell-expressed myc-p300(1-595). **D.** p300 knockdown substantially reduced the extent of ERβ ubiquitination in HEK293T cells.

Emerging evidence from studies of eukaryotic transcription suggests a mechanistic coupling between transcriptional activation and ubiquitin-dependent degradation of transcription activators by proteasomes [37, 38]. Consistent with such a notion, AF1 of ERβ has been implicated in ERβ ubiquitination and proteasome-mediated degradation [39, 40]. We therefore asked whether pY36 played a role in transcription-coupled ERβ ubiquitination and turnover. Our previous work indicated that the transcription coactivator p300 is recruited to ERβ target promoters, including that of MDA7, by ERβ in a pY36-dependent manner [34]. Here we found that ectopic expression of p300 promoted ligand-dependent transcriptional activation of ERβ-specific target gene MDA7 by ectopic ERβ in HEK293T cells (Figure 2A), whereas siRNA-mediated p300 knockdown blunted ERβ activity (Figure 2B). These data further confirm functional importance of p300 in potentiating ERβ transcriptional activity. Given the previously reported E4 ubiquitin ligase activity of p300 [41], we used an *in vitro* ubiquitination assay to assess the ability of p300 to ubiquitinate ERβ. In the presence of ubiquitin and E1/E2 ubiquitin ligases, ERβ was ubiquitinated by p300 expressed in mammalian cells, but not bacteria (lanes 4 and 5, Figure 2C). This could be due to posttranslational modification of p300 in mammalian cells required for ERβ ubiquitination. Alternatively, the observed ERβ ubiquitination could result from the combined action of p300 E4 and a coimmuniprecipitated E3 ubiqutin ligase. We next examined the effect of p300 on ERβ ubiquitination *in vivo*. We found that p300 knockdown substantially reduced the extent of Flag-tagged ERβ ubiquitination (compare lanes 2 and 4 in Figure 2D). Furthermore, WT ERβ was ubiquitinated more extensively than the Y36F mutant (compare lanes 2 and 6 in Figure 2D). This result strongly suggests that the phosphotyrosine switch promotes p300-mediated ERβ ubiquitination.

**Figure 3:**
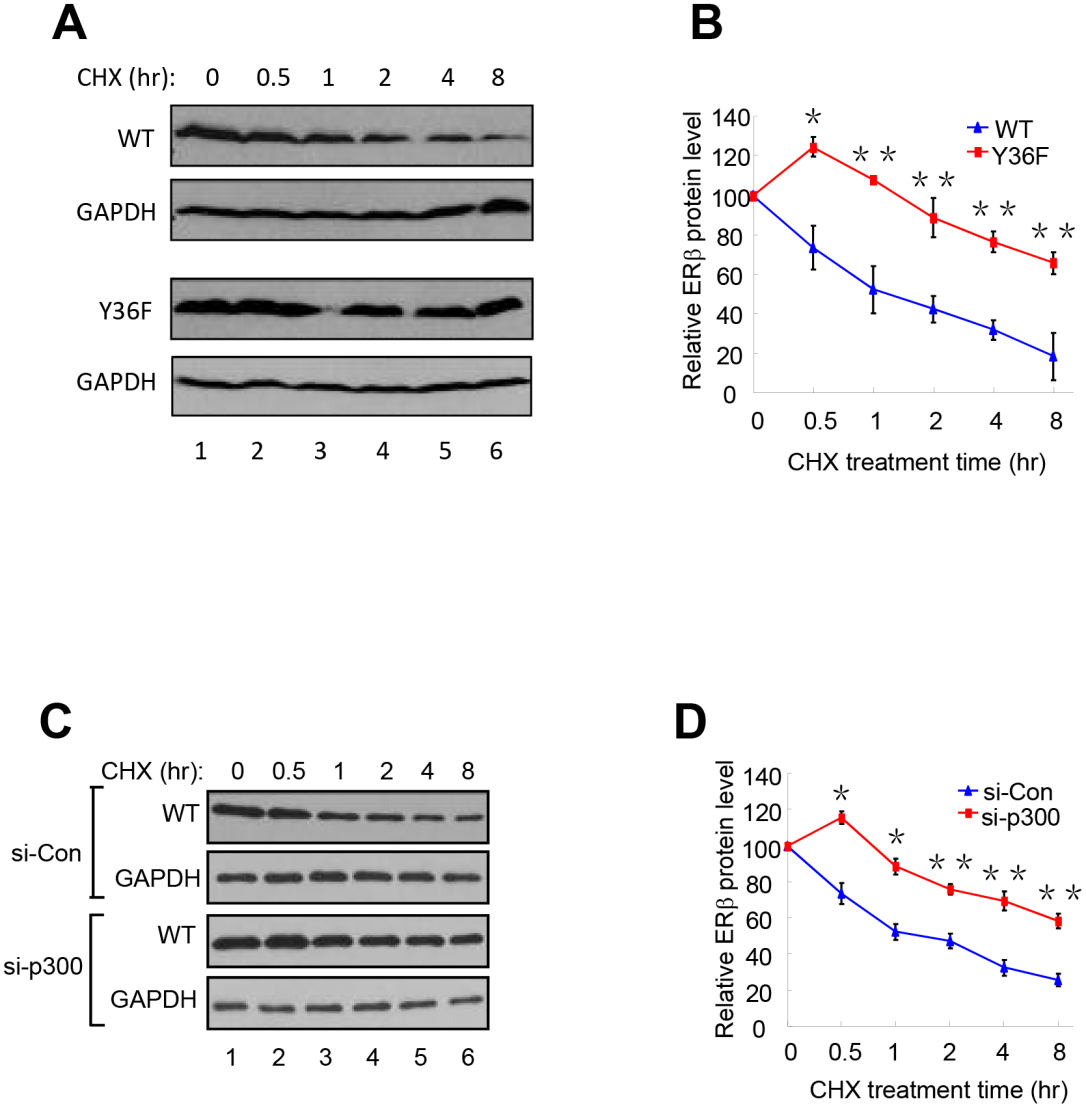
pY36 facilitates ERβ protein turnover **A.** and **B.** The half-lives of WT-ERβ and Y36F-mutant proteins were analyzed. HEK293T cells were transfected with plasmids encoding WT-ERβ or Y36F mutant. Cells were treated with cycloheximide (CHX) 24 h after transfection and collected at the indicated time points. The results were quantitated using Image J software. **C.** and **D.** WT-ERβ plasmid was co-transfected with si-Con or si-p300 oligos into HEK293T cells for 48 h. Transfected cells were subsequently treated with CHX for the indicated time.

Consistent with a role of proteasome-dependent regulation in ERβ protein turnover, we reproducibly observed stabilization of Flag-tagged WT ERβ by the treatment of proteasome inhibitor MG132 (compare lanes 1 and 2 in Figure 2D). To directly compare the half-life of WT and mutant ERβ protein, we assessed their abundance in the presence of the protein synthesis inhibitor cycloheximide. The Y36F mutant was substantially more stable (half-life > 8 hours, Figure 3A–3B) than its WT counterpart (approximately 1 hour). In a parallel experiment, p300 knockdown also significantly extended WT ERβ half-life (Figure 3C–3D). Together with our previous findings of pY36-dependent promoter recruitment of p300 by ERβ, our current data strongly indicate that the phosphotyrosine switch promotes two reciprocal events in ERβ-mediated transcriptional activation: p300 recruitment by ERβ and subsequent p300-dependent destruction of ERβ.

### Endogenous ERβ exhibits antitumor activity

**Figure 4:**
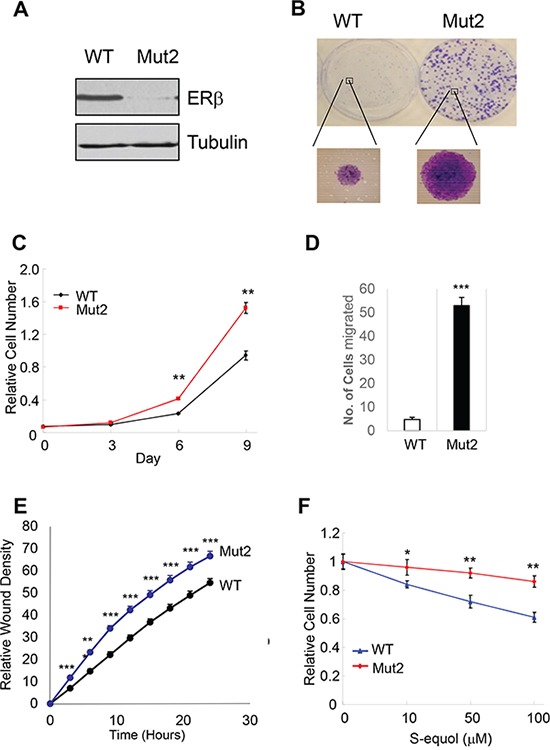
Endogenous ERβ exhibits antitumor activity **A.** CRISPR/Cas9 genome editing of ERβ-encoding ESR2 gene in MDA-MB-231 breast cancer cells. Western blot of ERβ in MDA-MB-231 cells and one ERβ-edited clone (Mut2). **B.** ERβ KO cells have enhanced colony-forming ability versus parental cells. **C.** ERβ KO cells exhibit accelerated cell growth. **D.** KO cells displays increased migratory ability in a Boyden chamber assay. **E.** Increased cell migration as assessed in a wound-healing assay. **F.** ERβ KO cells are more refractory to ERβ-selective agonist S-equol than parental cells. * p<0.05, **p<0.01.***p<0.001.

ERβ is expressed in a significant percentage of breast tumors across all subtypes [42, 43], making it a potential target in anticancer therapies. In addition, several natural and synthetic ERβ-selective agonists were well tolerated in clinical trials [44] (ClinicalTrials.gov), further elevating therapeutic feasibility of rallying ERβ antitumor activity. However, before any ERβ-targeting therapeutic agents are to be further explored for their clinical utility, it is important to verify their dependence on the presumed therapeutic target. To this end, we used CRISPR-Cas9 to knock out ERβ in triple-negative breast cancer cells MDA-MB-231. Several out-of-frame mutant clones were identified, two of which were selected for functional studies. The two clones contain an insertion of nucleotide “A” (Mut1) and deletion of “ACAA” (Mut2), respectively, at the engineered double strand break in the first protein-coding exon of the *ESR2* locus (Supplementary Figure S2A). Depletion of ERβ protein in both cell clones was confirmed by immunoblotting (Figure 4A and Supplementary Figure S2B). The resulting knockout cells grew significantly faster than the isogenic ERβ-expressing cells (Figure 4B–4C, Supplementary Figure S2C). In both Boyden chamber and wound-healing assays, the knockout cells also exhibited enhanced migratory ability versus the control cells (Figure 4D–4E). Furthermore, compared to the parental cells, ERβ knockout cells were more refractory to a previously characterized ERβ-selective agonist S-equol [18] (Figure 4F), thus supporting the selectivity of the ERβ-targeting compound.

### S-equol stimulates pY36 and inhibits tumor growth *in vivo*

**Figure 5:**
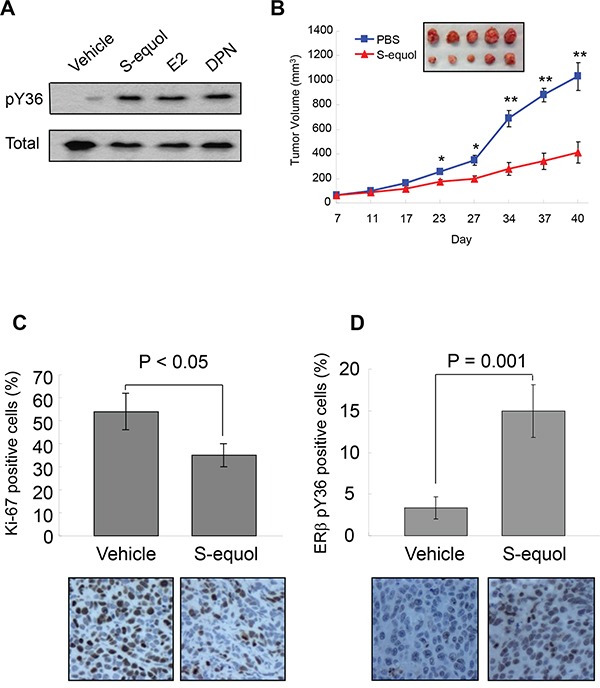
S-equol stimulates pY36 and inhibits tumor growth *in vivo* **A.** pY36-specific phosphorylation signal was enhanced by the ERα/ERβ common agonist 17-β-estradiol and two ERβ-specific agonists DPN and S-equol in MDA-MB-231 cells. **B.** S-equol treatment inhibited MDA-MB-231 cell-derived xenograft tumor growth (n = 5). **C.** Expression of Ki-67 in xenograft tumors. **D.** ERβ-pY36 signal in vehicle- and S-equol-treated xenograft tumor samples.* p<0.05, **p<0.01.

To explore the translational potential of the ERβ phosphotyrosine switch, we asked whether ERβ ligand could alter the pY36 status. As shown in Figure 5A, Y36-specific ERβ phosphorylation was enhanced by the ERα/ERβ common agonist 17β-estradiol and two ERβ-specific agonists DPN and S-equol, suggesting a crosstalk between the C-terminal ligand-binding domain and N-terminal phosphorylation site of ERβ. Furthermore, in a xenograft tumor model, S-equol inhibited MDA-MB-231 cell-derived tumor growth (Figure 5B), accompanied by significant reduction in the number of tumor cells expressing Ki-67, an established marker for cycling cells (Figure 5C), and with concomitant increase in pY36 signal in xenografted tumors (Figure 5D). This *in vivo* finding provides additional supporting evidence for a tumor-intrinsic function of the ERβ phosphotyrosine switch in therapeutic response to ERβ-activating compounds.

## DISCUSSION

Mobilization of ERβ antitumor activity is an attractive therapeutic strategy. Our current study bolsters the concept that rallying the antitumor activity of ERβ through its phosphotyrosine switch is applicable to both ERα-positive and -negative tumor cells of various cancer types. While our work focuses on the phosphotyrosine switch in full-length ERβ (ERβ1), we are aware of ERβ splicing variants (ERβ2-5), which share the same ERβ AF1 domain containing the phosphotyrosine residue yet have distinct biological activities from ERβ1. It will be important to determine in future studies whether the same phosphotyrosine switch regulates the functions of these variants.

**Figure 6:**
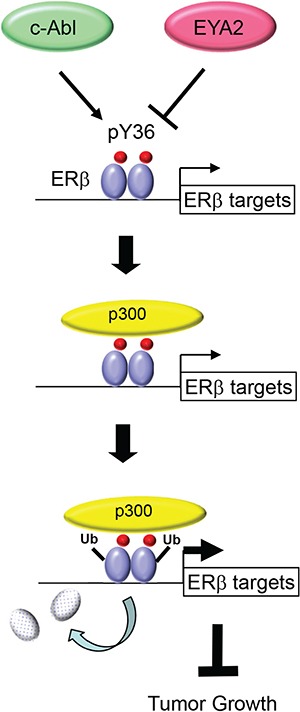
Model for transcription-coupled ERβ degradation

Our current work significantly extends mechanistic insights into the pY36-dependent regulation of ERβ transcriptional activity. In particular, the dual effects of the phosphotyrosine switch on ERβ-mediated transcriptional activation and ERβ turnover is reminiscent of accumulating reports of transcription-coupled ubiquitination and proteasome-mediated degradation of transcription factors [37, 38]. For example, the turnover of both ERα and its coactivator SRC3/AIB1 is coupled to their actions in transcriptional activation [45–47]. It remains to be determined whether pY36-dependent ubiquitination of ERβ occurs on chromatin and subsequent to coactivator recruitment. However, we favor the promoter-specific model, as Y36F mutation or overexpression of its corresponding phosphatase EYA2 increases chromatin occupancy of ERβ at ERβ-specific target promoters [34]. Such an activation-suicide process could ensure a rapid response of the transcription apparatus to fluctuating levels of estrogens and ERβ-specific stimuli [37, 38]. Alternatively, destruction of ERβ may be an obligatory part of transcriptional activation, without which the apparatus would be prevented from proceeding to the subsequent steps of transcription [38]. Regardless of the extent of transcription-ubiquitination coupling, the pY36-dependent functional reciprocity between ERβ and its coactivators likely results in active transcription of ERβ target genes and ultimately inhibition of tumor cell growth (Figure 6).

A number of clinically safe natural and synthetic compounds that function as ERβ-selective agonists have been identified [48]. For example, a synthetic ERβ-selective agonist LY500307 is being clinically tested for benign prostatic hyperplasia (BPH) and schizophrenia [49]. In addition, S-equol is clinically safe and well tolerated in humans, based on multiple phase I and II clinical trials [50–53]. As these compounds likely have additional targets *in vivo* besides ERβ, it is critical to distinguish their ERβ-dependent and -independent effects. The differential effects of S-equol on the proliferation of parental and ERβ knockout tumor cells observed in the current work strongly suggest that the antiproliferative action of S-equol is at least partly mediated by ERβ. Our findings are also consistent with previously reported antitumor effect of S-equol on MCF7-derived xenografted tumors [54]. Of note, stimulation of ERβ phosphorylation by S-equol observed both *in vitro* and *in vivo* raises the distinct possibility of crosstalk between ligand- and posttranslational modification-dependent activation of ERβ. While the exact mechanism underlying the N- (AF1) and C-terminal (AF2) crosstalk awaits future investigation, we speculate that this could be mediated by transcriptional coactivators that interact with both ERβ activation domains. Of note, previous studies of ERα suggest functional communications between AF1 and AF2 activation domains in ERα-mediated transcriptional activation and biological functions *in vivo* [55–60]. We therefore envision that simultaneous targeting of ERβ tyrosine phosphorylation and ligand binding could achieve maximal activity of ERβ for treating those ERβ-expressing cancers with an intact pY36 signaling circuitry. In summary, ERβ-selective agonists, together with the known kinase and phosphatase targeting this switch, provide multiple novel and druggable targets for activating the subtype-specific function of ERβ.

## MATERIALS AND METHODS

### Cell lines and reagents

The expression vectors for WT and mutant ERβ were described previously [34]. The plasmid for p300 expression was kindly provided by Dr. Zhi-Min Yuan (Harvard T.H. Chan School of Public Health). Cell lines were originally purchased from the American Type Culture Collection and cultured per their instructions.17-β-estradiol (E2), DPN, and MG132 were obtained from Tocris, Inc. S-equol was generously provided by Ausio Pharmaceuticals. The following antibodies were purchased commercially: anti-Flag M2 (A8592 and F3165, Sigma-Aldrich), anti-ERβ for immunoblotting (14C8, GeneTex; 9.88, Abcam), anti-ERβ for immunoprecipitation (IP) (EPR3777, Novus), anti-p300 (sc-584, Santa Cruz Biotechnology Inc.), anti-GAPDH (G9295, Sigma-Aldrich), anti-FLAG-HRP (A8592, Sigma-Aldrich), anti-Flag M2 agarose (A2220, Sigma-Aldrich), and anti-Ki67 (GTX16667, GeneTex). The rabbit polyclonal anti-pY36 antibody was raised as previously described [34]. Oligonucleotides si-Con (non-targeting) (L-001810-10), si-p300 (L-003486-00), ON-TARGETplus smart pool siRNA duplexes were purchased from Dharmacon.

### MTT assay

Cell viability was measured by the MTT (3-(4,5-dimethylthiazol-2-yl)-2,5-diphenyltetrazolium bromide) using the manufacturer's protocol (ATCC). Briefly, cells were plated in 96-well microtiter plates, allowed to attach overnight, and treated with concentrations of S-equol as indicated for 72 h at 37°C. MTT was added to the culture medium to yield a final concentration of 0.5 mg/ml following cell treatment, and the incubation was continued for 1 h at 37°C. The pellets were dissolved with dimethyl sulfoxide at room temperature for 10 min. Cell viability was determined by measuring the absorbance of the converted dye at a wave length of 570 nm.

### Cell migration/invasion assays

For assessing MCF7 cell migration, cells were grown to 80% confluence and harvested from the plate using 0.05% trypsin-EDTA. Cells were collected, washed with serum-free medium twice, and resuspended in serum-free medium. Twenty-four-well transwell chambers (BD BioCoat Cat.# 354575) with 8.0 μm pore size polycarbonate membranes were used. Cells were plated at 1 × 10^5^ cells/well in 0.5 mL in the inserts, which were then placed into chambers containing growth medium with 10% FBS. After incubation at 37°C, for 24 h, inserts were removed and cells were fixed in 4% paraformaldehyde and stained with 0.1% crystal-violet. Cells on the upper membrane surface were removed with a cotton swab. Air-dried membranes were viewed under 10 x magnification and migrated cells were counted in five randomly chosen fields/membrane. Each cell line was assayed at least 3 times and assays were performed in duplicate. Error bars show s.e.m. For invasion assays, Matrigel-coated inserts with 8.0 μm pore size membranes (BD BioCoat Cat.# 354480) were used.

For assessing MDA-MB-231 cell migration, cells were grown to 80% confluency, serum-starved for 3 h, and then harvested from the plate using 0.05% trypsin-EDTA. Cells were seeded at 5 × 10^4^ cells/200 μl of serum-free medium per insert, which were then placed into 24-well transwell chambers (BD BioCoat Cat.# 354575, 8.0 μm pore size), containing growth medium with 10% FBS. After incubation at 37°C for 4 h, the cells were fixed and processed as mentioned above. For the wound-healing assay, cells were seeded in 96-well image lock plate (Essen BioScience Cat.#4379) in triplicates, at 70,000 cells/well in growth medium with 10% FBS. After incubation at 37°C for 6 h, a scratch was made and wound healing was assessed in time lapse for 24 h using IncuCyte Live-Cell Imaging System (IncuCyte ZOOM®, Essen Bioscience Inc.).

### *In vitro* ubiquitination assay

Recombinant His-ERβ or GST-p300(1-595) was constructed, expressed and purified from *E.coli* BL21 cells expressing PET32a-ERβ or GST-p300(1-595) according to the manufactures' instructions (Qiagen and Amersham). Myc-p300(1-595) protein immunoprecipitates were immunoprecipitated with Myc beads from 293T cells transfected with Myc-p300(1-595) and eluted with a Myc peptide. *In vitro* ubiquitination assays were performed in ubiquitination reaction buffer (25 mM Hepes pH 7.4, 10 mM NaCl, 3 mM MgCl2, 0.05% Triton X-100, 0.5 mM DTT, 3 mM Mg-ATP (B-20, Boston Biochem)) with 100 ng E1 (E-305, Boston Biochem), 50 ng E2 (ubch5a, E2-616, Boston Biochem), 5 μg ubiquitin (Ub, U-100Pf, Boston Biochem), and were incubated for 60 min at 37°C. The samples were subjected to SDS-PAGE and analyzed with indicated antibodies.

### *In vivo* ubiquitination assay

293T cells were transfected with WT or mutant ERβ. Twenty-four h after transfection, cells were re-seeded and transfected with control siRNA or p300 siRNA with Lipofectamine RNAiMAX transfection reagent (Invitrogen). Two days following transfection, cells were treated with MG132 (5 μM) for 6 h. Immunoprecipitation was performed as previously described [61].

### Protein half-life assay

HEK293T cells were transfected with WT and mutant ERβ. 24 h after transfection, fresh medium containing cycloheximide (Sigma) was added to a final concentration of 50 μM. Cells were harvested at indicated time points. ERβ steady-state levels were analyzed by Western blotting. Each result was derived from at least three independent experiments assessing densitometry-based protein ERβ quantification using GAPDH as the internal control.

### Ligand treatment

For ligand stimulation, cells were cultured in phenol red-free medium containing 5% charcoal stripped (CS) FBS for 3 days, re-seeded in Nunclon plates, and transfected with various vectors as indicated with Lipofectamine 2000 (Invitrogen). Six h after transfection, cells were treated with either vehicle or ligand at the indicated final concentration.

### Generation of ERβ CRISPR knockout cells

ERβ specific sgRNA target sequences were cloned into the CRISPR v2 vector (Addgene plasmid #52961). The 20-bp target sequences of the indicated sgRNAs were as follows: sgERβ-1, GGATTGACTGCAGTTGTAGG; sgERβ-2, GAAGGAGAATTAAGGCTAGA. Three days after transfection, cells were selected with puromycin (Sigma) at 1 μg/ml for 3 weeks. Genomic DNA was extracted using the PureLink Genomic DNA Mini Kit (K1820-00, Life Technologies) according to the manufacturer's instructions. Drug-resistant cell clones were propagated and screened for mutations at nuclease target sites by PCR amplification of genomic sequences followed by DNA sequencing.

### Xenografts

All animal experiments were performed after obtaining University od Texas Health Science Center at San Antonio (UTHSCSA) IACUC approval, and all methods were carried out in accordance with the IACUC approved guidelines. 5 × 10^6^ MDA-MB-231 cells were injected orthotopically into mammary gland fat pads of 6 week-old female athymic nude mice (Harlan). When the tumor masses reached 50 to 80 mm^3^ (about one week after the inoculation), the mice were given daily subcutaneous injections of S-equol (20 mg/kg per day) or PBS as a vehicle control. Tumor development was followed by caliper measurements along two orthogonal axes: length (L) and width (W) and volume (V) was estimated by the formula V = [L x (W^2^)]/2.

### Immunohistochemistry

Xenograft tumors harvested from mice were fixed in 10% neutral-buffered formalin, dehydrated, embedded in paraffin, and sectioned at 3 μm thickness. Representative tumor sections from vehicle control and S-equol-treated mice were tested for Ki-67 expression to assess cell proliferation, and for ERβ pY36.

### Statistics

Statistical significance in the experiments was assessed by two-tailed Student's *t* test. In all assays, p < 0.05 was considered statistically significant.

## SUPPLEMENTARY MATERIALS FIGURES



## References

[1] Thomas C, Gustafsson JA (2011). The different roles of ER subtypes in cancer biology and therapy. Nat Rev Cancer.

[2] Bossard C, Busson M, Vindrieux D, Gaudin F, Machelon V, Brigitte M, Jacquard C, Pillon A, Balaguer P, Balabanian K, Lazennec G (2012). Potential role of estrogen receptor beta as a tumor suppressor of epithelial ovarian cancer. PloS one.

[3] Cho JL, Allanson M, Reeve VE (2010). Oestrogen receptor-beta signalling protects against transplanted skin tumour growth in the mouse. Photochem Photobiol Sci.

[4] Sareddy GR, Nair BC, Gonugunta VK, Zhang QG, Brenner A, Brann DW, Tekmal RR, Vadlamudi RK (2012). Therapeutic significance of estrogen receptor beta agonists in gliomas. Mol Cancer Ther.

[5] Hodges-Gallagher L, Valentine CD, El Bader S, Kushner PJ (2008). Estrogen receptor beta increases the efficacy of antiestrogens by effects on apoptosis and cell cycling in breast cancer cells. Breast Cancer Res Treat.

[6] Strom A, Hartman J, Foster JS, Kietz S, Wimalasena J, Gustafsson JA (2004). Estrogen receptor beta inhibits 17beta-estradiol-stimulated proliferation of the breast cancer cell line T47D. Proc Natl Acad Sci U S A.

[7] Hartman J, Lindberg K, Morani A, Inzunza J, Strom A, Gustafsson JA (2006). Estrogen receptor beta inhibits angiogenesis and growth of T47D breast cancer xenografts. Cancer Res.

[8] Monroe DG, Getz BJ, Johnsen SA, Riggs BL, Khosla S, Spelsberg TC (2003). Estrogen receptor isoform-specific regulation of endogenous gene expression in human osteoblastic cell lines expressing either ERalpha or ERbeta. J Cell Biochem.

[9] Kian Tee M, Rogatsky I, Tzagarakis-Foster C, Cvoro A, An J, Christy RJ, Yamamoto KR, Leitman DC (2004). Estradiol and selective estrogen receptor modulators differentially regulate target genes with estrogen receptors alpha and beta. Mol Biol Cell.

[10] Stossi F, Barnett DH, Frasor J, Komm B, Lyttle CR, Katzenellenbogen BS (2004). Transcriptional profiling of estrogen-regulated gene expression via estrogen receptor (ER) alpha or ERbeta in human osteosarcoma cells: distinct and common target genes for these receptors. Endocrinology.

[11] Secreto FJ, Monroe DG, Dutta S, Ingle JN, Spelsberg TC (2007). Estrogen receptor alpha/beta isoforms but not betacx modulate unique patterns of gene expression and cell proliferation in Hs578T cells. J Cell Biochem.

[12] Hawse JR, Subramaniam M, Monroe DG, Hemmingsen AH, Ingle JN, Khosla S, Oursler MJ, Spelsberg TC (2008). Estrogen receptor beta isoform-specific induction of transforming growth factor beta-inducible early gene-1 in human osteoblast cells: an essential role for the activation function 1 domain. Mol Endocrinol.

[13] Paruthiyil S, Cvoro A, Zhao X, Wu Z, Sui Y, Staub RE, Baggett S, Herber CB, Griffin C, Tagliaferri M, Harris HA, Cohen I, Bjeldanes LF, Speed TP, Schaufele F, Leitman DC (2009). Drug and cell type-specific regulation of genes with different classes of estrogen receptor beta-selective agonists. PLoS One.

[14] Chang EC, Frasor J, Komm B, Katzenellenbogen BS (2006). Impact of estrogen receptor beta on gene networks regulated by estrogen receptor alpha in breast cancer cells. Endocrinology.

[15] Charn TH, Liu ET, Chang EC, Lee YK, Katzenellenbogen JA, Katzenellenbogen BS (2010). Genome-wide dynamics of chromatin binding of estrogen receptors alpha and beta: mutual restriction and competitive site selection. Mol Endocrinol.

[16] Vivar OI, Zhao X, Saunier EF, Griffin C, Mayba OS, Tagliaferri M, Cohen I, Speed TP, Leitman DC (2010). Estrogen receptor beta binds to and regulates three distinct classes of target genes. J Biol Chem.

[17] Zhao C, Gao H, Liu Y, Papoutsi Z, Jaffrey S, Gustafsson JA, Dahlman-Wright K (2010). Genome-wide mapping of estrogen receptor-beta-binding regions reveals extensive cross-talk with transcription factor activator protein-1. Cancer Res.

[18] Jiang Y, Gong P, Madak-Erdogan Z, Martin T, Jeyakumar M, Carlson K, Khan I, Smillie TJ, Chittiboyina AG, Rotte SC, Helferich WG, Katzenellenbogen JA, Katzenellenbogen BS (2013). Mechanisms enforcing the estrogen receptor beta selectivity of botanical estrogens. FASEB J.

[19] Shanle EK, Zhao Z, Hawse J, Wisinski K, Keles S, Yuan M, Xu W (2013). Research resource: global identification of estrogen receptor beta target genes in triple negative breast cancer cells. Mol Endocrinol.

[20] Sun J, Meyers MJ, Fink BE, Rajendran R, Katzenellenbogen JA, Katzenellenbogen BS (1999). Novel ligands that function as selective estrogens or antiestrogens for estrogen receptor-alpha or estrogen receptor-beta. Endocrinology.

[21] Paruthiyil S, Parmar H, Kerekatte V, Cunha GR, Firestone GL, Leitman DC (2004). Estrogen receptor beta inhibits human breast cancer cell proliferation and tumor formation by causing a G2 cell cycle arrest. Cancer Res.

[22] Williams C, Edvardsson K, Lewandowski SA, Strom A, Gustafsson JA (2008). A genome-wide study of the repressive effects of estrogen receptor beta on estrogen receptor alpha signaling in breast cancer cells. Oncogene.

[23] Frasor J, Chang EC, Komm B, Lin CY, Vega VB, Liu ET, Miller LD, Smeds J, Bergh J, Katzenellenbogen BS (2006). Gene expression preferentially regulated by tamoxifen in breast cancer cells and correlations with clinical outcome. Cancer Res.

[24] Chang EC, Charn TH, Park SH, Helferich WG, Komm B, Katzenellenbogen JA, Katzenellenbogen BS (2008). Estrogen Receptors alpha and beta as determinants of gene expression: influence of ligand dose and chromatin binding. Mol Endocrinol.

[25] Deroo BJ, Buensuceso AV (2010). Minireview: Estrogen receptor-beta: mechanistic insights from recent studies. Mol Endocrinol.

[26] Pettersson K, Grandien K, Kuiper GG, Gustafsson JA (1997). Mouse estrogen receptor beta forms estrogen response element-binding heterodimers with estrogen receptor alpha. Mol Endocrinol.

[27] Cowley SM, Hoare S, Mosselman S, Parker MG (1997). Estrogen receptors alpha and beta form heterodimers on DNA. J Biol Chem.

[28] Tremblay GB, Tremblay A, Labrie F, Giguere V (1999). Dominant activity of activation function 1 (AF-1) and differential stoichiometric requirements for AF-1 and −2 in the estrogen receptor alpha-beta heterodimeric complex. Mol Cell Biol.

[29] Hall JM, McDonnell DP (1999). The estrogen receptor beta-isoform (ERbeta) of the human estrogen receptor modulates ERalpha transcriptional activity and is a key regulator of the cellular response to estrogens and antiestrogens. Endocrinology.

[30] Li X, Huang J, Yi P, Bambara RA, Hilf R, Muyan M (2004). Single-chain estrogen receptors (ERs) reveal that the ERalpha/beta heterodimer emulates functions of the ERalpha dimer in genomic estrogen signaling pathways. Mol Cell Biol.

[31] Paulmurugan R, Tamrazi A, Massoud TF, Katzenellenbogen JA, Gambhir SS (2011). In vitro and in vivo molecular imaging of estrogen receptor alpha and beta homo- and heterodimerization: exploration of new modes of receptor regulation. Mol Endocrinol.

[32] Smith CL, O'Malley B (2004). Coregulator function: a key to understanding tissue specificity of selective receptor modulators. Endocr Rev.

[33] Madak-Erdogan Z, Charn TH, Jiang Y, Liu ET, Katzenellenbogen JA, Katzenellenbogen BS (2013). Integrative genomics of gene and metabolic regulation by estrogen receptors alpha and beta and their coregulators. Mol Syst Biol.

[34] Yuan B, Cheng L, Chiang HC, Xu X, Han Y, Su H, Wang L, Zhang B, Lin J, Li X, Xie X, Wang T, Tekmal RR, Curiel TJ, Yuan ZM, Elledge R (2014). A phosphotyrosine switch determines the antitumor activity of ERbeta. J Clin Invest.

[35] Gallo D, De Stefano I, Grazia Prisco M, Scambia G, Ferrandina G (2012). Estrogen receptor beta in cancer: an attractive target for therapy. Curr Pharm Des.

[36] Lam HM, Suresh Babu CV, Wang J, Yuan Y, Lam YW, Ho SM, Leung YK (2012). Phosphorylation of human estrogen receptor-beta at serine 105 inhibits breast cancer cell migration and invasion. Molecular and cellular endocrinology.

[37] Conaway RC, Brower CS, Conaway JW (2002). Emerging roles of ubiquitin in transcription regulation. Science.

[38] Geng F, Wenzel S, Tansey WP (2012). Ubiquitin and proteasomes in transcription. Annual review of biochemistry.

[39] Tateishi Y, Sonoo R, Sekiya Y, Sunahara N, Kawano M, Wayama M, Hirota R, Kawabe Y, Murayama A, Kato S, Kimura K, Yanagisawa J (2006). Turning off estrogen receptor beta-mediated transcription requires estrogen-dependent receptor proteolysis. Molecular and cellular biology.

[40] Picard N, Charbonneau C, Sanchez M, Licznar A, Busson M, Lazennec G, Tremblay A (2008). Phosphorylation of activation function-1 regulates proteasome-dependent nuclear mobility and E6-associated protein ubiquitin ligase recruitment to the estrogen receptor beta. Mol Endocrinol.

[41] Grossman SR, Deato ME, Brignone C, Chan HM, Kung AL, Tagami H, Nakatani Y, Livingston DM (2003). Polyubiquitination of p53 by a ubiquitin ligase activity of p300. Science.

[42] Marotti JD, Collins LC, Hu R, Tamimi RM (2010). Estrogen receptor-beta expression in invasive breast cancer in relation to molecular phenotype: results from the Nurses' Health Study. Modern Pathology.

[43] Reese JM, Suman VJ, Subramaniam M, Wu X, Negron V, Gingery A, Pitel KS, Shah SS, Cunliffe HE, McCullough AE, Pockaj BA, Couch FJ, Olson JE, Reynolds C, Lingle WL, Spelsberg TC (2014). ERbeta1: characterization prognosis and evaluation of treatment strategies in ERalpha-positive and -negative breast cancer. BMC Cancer.

[44] Nilsson S, Koehler KF, Gustafsson JA (2011). Development of subtype-selective oestrogen receptor-based therapeutics. Nat Rev Drug Discov.

[45] Lonard DM, Nawaz Z, Smith CL, O'Malley BW (2000). The 26S proteasome is required for estrogen receptor-alpha and acoactivator turnover and for efficient estrogen receptor-alpha transactivation. Mol Cell.

[46] Shao W, Keeton EK, McDonnell DP, Brown M (2004). Coactivator AIB1 links estrogen receptor transcriptional activity and stability. Proceedings of the National Academy of Sciences of the United States of America.

[47] Li C, Liang YY, Feng XH, Tsai SY, Tsai MJ, O'Malley BW (2008). Essential phosphatases and a phospho-degron are critical for regulation of SRC-3/AIB1 coactivator function and turnover. Molecular cell.

[48] Warner M, Gustafsson JA (2010). The role of estrogen receptor beta (ERbeta) in malignant diseases–a new potential target for antiproliferative drugs in prevention and treatment of cancer. Biochem Biophys Res Commun.

[49] Roehrborn CG, Spann ME, Myers SL, Serviss CR, Hu L, Jin Y (2015). Estrogen receptor beta agonist LY500307 fails to improve symptoms in men with enlarged prostate secondary to benign prostatic hypertrophy. Prostate Cancer Prostatic Dis.

[50] Setchell KD, Clerici C, Lephart ED, Cole SJ, Heenan C, Castellani D, Wolfe BE, Nechemias-Zimmer L, Brown NM, Lund TD, Handa RJ, Heubi JE (2005). S-equol a potent ligand for estrogen receptor beta is the exclusive enantiomeric form of the soy isoflavone metabolite produced by human intestinal bacterial flora. Am J Clin Nutr.

[51] Jackson RL, Greiwe JS, Schwen RJ (2011). Emerging evidence of the health benefits of S-equol an estrogen receptor beta agonist. Nutrition Rev.

[52] Ishiwata N, Melby MK, Mizuno S, Watanabe S (2009). New equol supplement for relieving menopausal symptoms: randomized placebo-controlled trial of Japanese women. Menopause.

[53] Jackson RL, Greiwe JS, Desai PB, Schwen RJ (2011). Single-dose and steady-state pharmacokinetic studies of S-equol a potent nonhormonal estrogen receptor beta-agonist being developed for the treatment of menopausal symptoms. Menopause.

[54] Onoda A, Ueno T, Uchiyama S, Hayashi S, Kato K, Wake N (2011). Effects of S-equol and natural S-equol supplement (SE5-OH) on the growth of MCF-7 in vitro and as tumors implanted into ovariectomized athymic mice. Food Chem Toxicol.

[55] Merot Y, Metivier R, Penot G, Manu D, Saligaut C, Gannon F, Pakdel F, Kah O, Flouriot G (2004). The relative contribution exerted by AF-1 and AF-2 transactivation functions in estrogen receptor alpha transcriptional activity depends upon the differentiation stage of the cell. J Biol Chem.

[56] Chen A, Kleiman FE, Manley JL, Ouchi T, Pan ZQ (2002). Autoubiquitination of the BRCA1*BARD1 RING ubiquitin ligase. J Biol Chem.

[57] Borjesson AE, Windahl SH, Lagerquist MK, Engdahl C, Frenkel B, Moverare-Skrtic S, Sjogren K, Kindblom JM, Stubelius A, Islander U, Antal MC, Krust A, Chambon P, Ohlsson C (2011). Roles of transactivating functions 1 and 2 of estrogen receptor-alpha in bone. Proc Natl Acad Sci U S A.

[58] Shah SP, Roth A, Goya R, Oloumi A, Ha G, Zhao Y, Turashvili G, Ding J, Tse K, Haffari G, Bashashati A, Prentice LM, Khattra J, Burleigh A, Yap D, Bernard V (2012). The clonal and mutational evolution spectrum of primary triple-negative breast cancers. Nature.

[59] Arao Y, Coons LA, Zuercher WJ, Korach KS (2015). Transactivation Function-2 of Estrogen Receptor alpha Contains Transactivation Function-1-regulating Element. J Biol Chem.

[60] Nwachukwu JC, Srinivasan S, Zheng Y, Wang S, Min J, Dong C, Liao Z, Nowak J, Wright NJ, Houtman R, Carlson KE, Josan JS, Elemento O, Katzenellenbogen JA, Zhou HB, Nettles KW (2016). Predictive features of ligand-specific signaling through the estrogen receptor. Mol Syst Biol.

[61] Aiyar SE, Sun J-L, Blair AL, Moskaluk CA, Lv Y, Ye Q-N, Yamaguchi Y, Mukherjee A, Ren D-M, Handa H, Li R (2004). Attenuation of estrogen receptor alpha-mediated transcription through estrogen-stimulated recruitment of a negative elongation factor. Genes & Dev.

