# Supplementary material for: Tyrosine phosphorylation regulates ERβ ubiquitination, protein turnover, and inhibition of breast cancer
Source: Oncotarget. 2016 Jun 14;7(27):42585–97. doi: 10.18632/oncotarget.10018 (PMC5173158; doi:10.18632/oncotarget.10018)
Supplement: Supplementary file 1 [file oncotarget-07-42585-s001.pdf]

Tyrosine phosphorylation regulates ERβ ubiquitination, protein turnover, and inhibition of breast cancer

SUPPLEMENTARY FIGURES

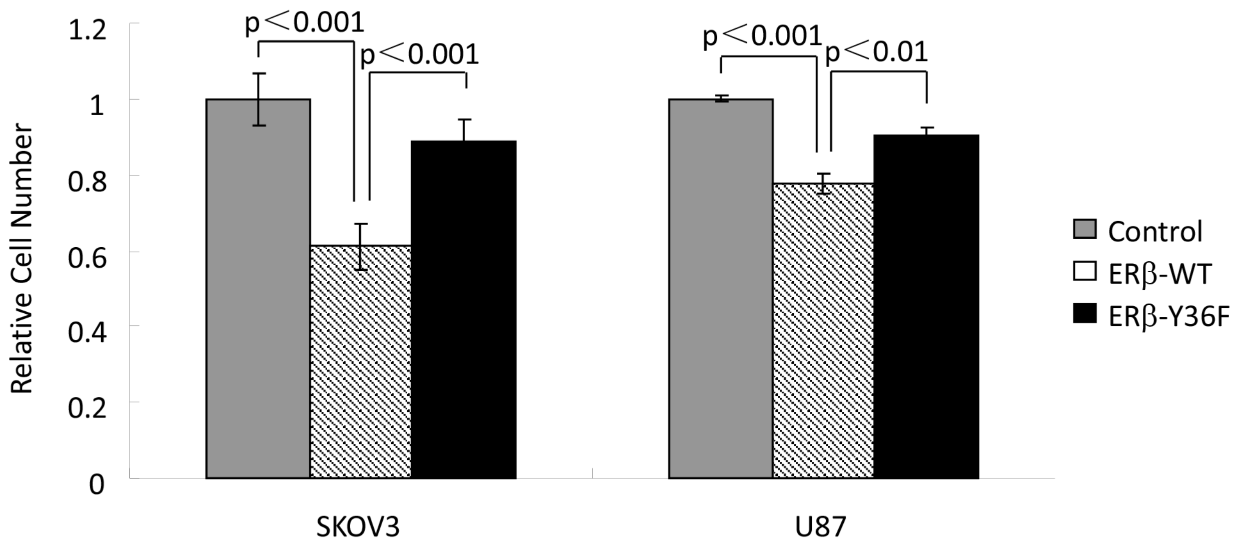

Supplementary Figure S1: MTT-based *in vitro* growth assay of SKOV3 ovarian cancer cells and U87 glioma cells transfected with vector alone, WT ERβ, and Y36F mutant ERβ.

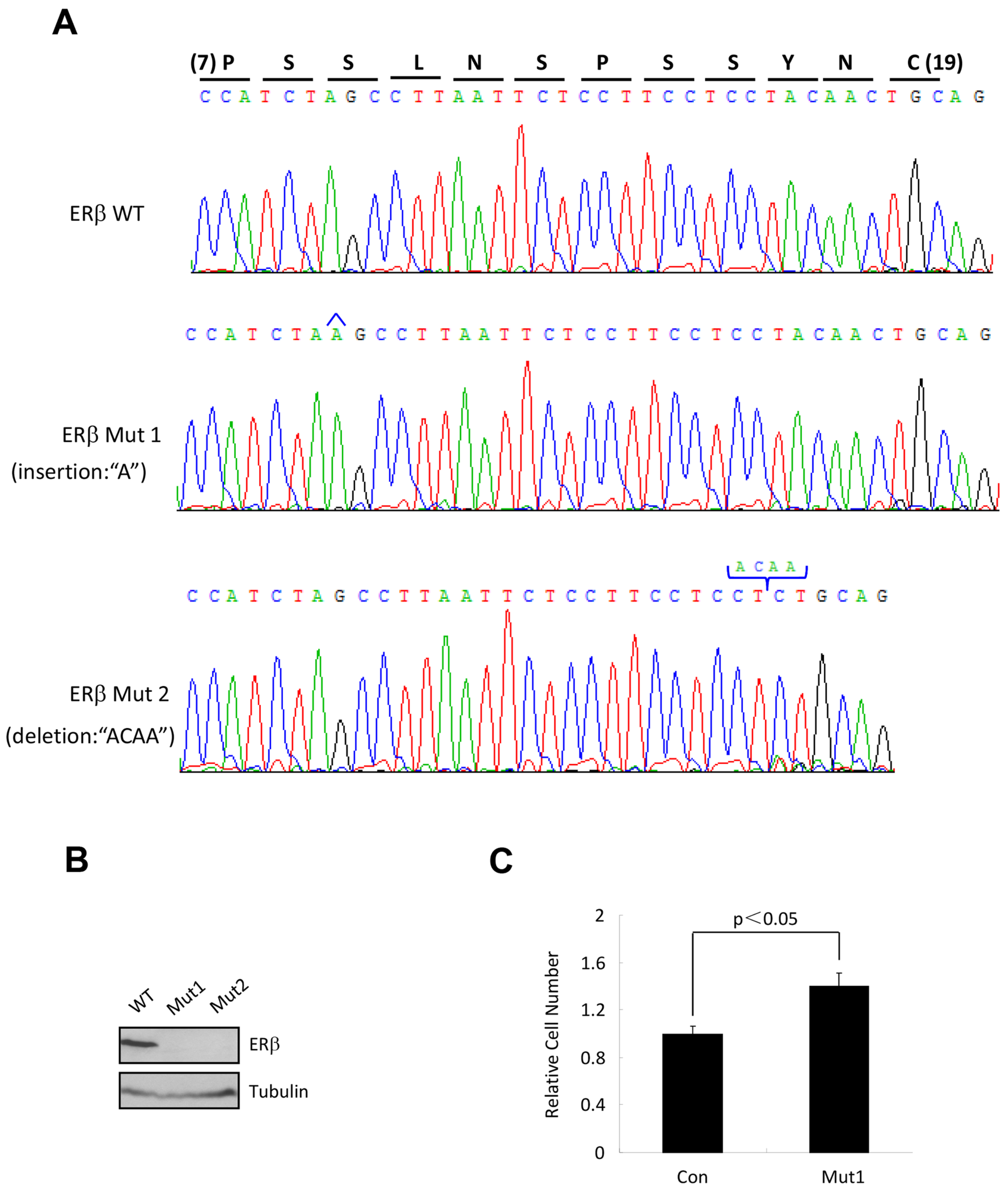

Supplementary Figure S2: *ESR2* knockout in MDA-MB-231 cells. A. DNA sequences of WT and mutant *ESR2*. **B.** Protein levels of WT and the two mutant cells derived from MDA-MB-231 cells. **C.** MTT-based growth assay for Mut1 cell clone.
